# Cardiothoracic surgery at a crossroads: The impact of disruptive technologic change

**DOI:** 10.1186/1749-8090-2-35

**Published:** 2007-08-08

**Authors:** David J Cohen

**Affiliations:** 1 Colonel, Medical Corps, United States Army (retired), President, Alamo Cardiothoracic Surgical Associates, PA San Antonio, TX, USA

## Abstract

At the beginning of the twenty-first century, cardiothoracic surgery is arguably the most successful of all medical specialties. There are effective treatments including transplantation, for almost all cardiac and thoracic diseases that can be performed with low morbidity and mortality. Cardiothoracic surgeons have mastered technical difficulties through innovation, hard work, planning and skill. Yet in the past decade, the primacy of cardiothoracic surgery has been challenged by new technologies. This paper applies business school theories to examine how cardiothoracic surgeons might best respond to such "disruptive technologies". Otherwise well-managed business and industrial enterprises have had difficulty dealing with disruptive technological change because of well-recognized organizational impediments. Cardiothoracic surgeons must understand the characteristics of disruptive technologies and consider organizational changes that will allow the profession to better adapt to them.

## Background

Throughout the latter half of the twentieth century, cardiothoracic surgeons have prided themselves as being among the most talented and sophisticated of all of their surgical colleagues. They not only mastered the field of general surgery, but they have had very long, difficult training, and they have charted new areas of research and technical expertise. Perhaps most importantly, they have performed extremely dangerous and sophisticated operations, with good results that have kept improving. If the specialty of cardiothoracic surgery were a unified organization, it would be considered well managed. As a discipline, cardiothoracic surgery has met the technical difficulties encountered through innovation, hard work, planning, and skill. Notwithstanding this record of performance, during the past decade cardiothoracic surgery has been challenged and many of its most successful procedures now are at risk of becoming obsolete by less elegant procedures with less certain outcomes performed by specialists with less training. This paper examines whether the specialty of cardiothoracic surgery is at risk of failing. It will use industrial models originally developed to explore the difficulties well managed companies faced when confronting new technologies. It is the premise of these models that there are two types of technologies: (1) sustaining technology, which is successfully mastered by established industries and (2) disruptive technology, which is problematic and poses a great challenge to established organizations.

Cardiothoracic surgery has evolved logically from its beginnings in general surgery to the surgery of pulmonary tuberculosis, then to lung cancer surgery and presently to open-heart surgery. As cardiothoracic surgery has become more technologically driven by cardiopulmonary bypass procedures and specifically coronary artery bypass graft surgery, it has demonstrated the characteristic changes of many established and successful enterprises as they have faced continuing technological changes. To understand what is happening to the profession of cardiothoracic surgery, it is necessary to examine how other disciplines have dealt and continue to deal with technological innovation and to develop analogies between the practice of cardiothoracic surgery and these different organizations. In particular, while coronary artery bypass graft surgery has successfully incorporated advances due to improvements in sustaining technology, it has also faced the threat of radical or disruptive technological change such as that posed by angioplasty and coronary artery stenting.

### Abernathy-Utterbach Model

James Utterback in his book Mastering the Dynamics of Innovation states that "innovation is at once the creator and destroyer of industries and corporations" [1]. Technological change is both a creative force in the growth of corporations and a destructive force making those same corporations vulnerable to competitors. "Today when competitiveness hinges on the ability to develop or adapt new technologies in products, services, and processes, understanding the dynamics of industrial innovation and change is essential for survival and success" [1]. This is as true for cardiothoracic surgery as it is for industry. Utterback and his colleague William Abernathy have developed a model describing the dynamics of innovation in industry which they validated using many industrial examples. The model can easily be applied to the development of cardiothoracic surgery.

Their model describes three phases of product and process innovation that form a general pattern. The first, known as the "**fluid phase**" occurs during an industry's or product's formative years. There is a great deal of experimentation with product design and operational characteristics by numerous competitors. An example which Utterback cites is the early period of the automobile industry when there was a bewildering array of car models and engines including 3 wheel cars, 4 wheel cars, motorized buggies, as well as steam, electric and finally gasoline propulsion. "During this fluid period of high *product innovation*, much less attention is given to the processes by which products are made, so the rate of *process innovation *is significantly less rapid" [2]. In cardiac surgery, this phase is easily identified as the early period when cross circulation techniques were being tried, cardiopulmonary bypass was invented, new vascular graft prostheses and valve prostheses were rapidly fielded and a wide variety of new operations for all manner of congenital and acquired cardiac defects were presented monthly in the journals.

This fluid period gives way to a "**transitional phase**" in which innovations in the basic product slow down and the focus shifts toward innovations in the process of making the product or performing the service. Variety in the product tends to give way to standard designs that have either proven themselves in the marketplace as the best form for satisfying user needs, or designs that have been dictated by accepted standards, by legal or by regulatory constraints. In the automobile industry, the dominant design that emerged was the steel frame automobile fully enclosed as a sedan or coupe, as first introduced by Dodge. Even today's automobiles are recognizable descendents of this dominant design. In coronary artery surgery, the operation performed on cardiopulmonary bypass using cardiac arrest with cold cardioplegia and any variety of conduits has emerged as the dominant design for over 3 decades. Professional standards and training, industry innovation, patient expectation, and the regulations of the Food and Drug Administration all contributed to this process, much as similar forces impacted the automobile industry.

A final phase which Abernathy and Utterback call the "**specific phase**" occurs when the rate of major innovation dwindles for both product development and for manufacturing process development. Not all industries enter this phase, but those that do become "extremely focused on cost, volume, and capacity. Product and process innovation appears in small, incremental steps" [2]. Organizational arrangements become important. Efficiency and effectiveness become the basis for competition. Some might think that cardiac surgery entered this phase with the advent of the managed care era with its emphasis predominately on price and efficiency, while others will feel that it is still in the midst of the transition phase.

Utterback also describes technological "discontinuities", which are disruptive technologies that reshuffle the deck of corporate and industrial leadership. The key observation that Utterback draws from his study is "the extent to which industries experience waves of change and innovation interspersed with periods of stability and consolidation. When a wave of radical innovation sweeps across an industry, by definition it renders one or more existing technologies obsolete, and the firms with products and internal capabilities bound up by those existing technologies must either get aboard the new one or expect to be swept away or relegated to some new role in the industry" [3]. As the specialty of cardiothoracic surgery has increasingly shifted from an anatomical, organ system basis to a specialty dominated by the performance of specific high volume procedures, it too has become subject to these same types of technological forces.

It is important to be aware of the several themes that Utterback recognizes. Some of these will also prove relevant in the case of cardiothoracic surgery, but even more specifically, cardiac surgery. First, "established firms must occasionally attempt to renew and diversify their core businesses rather than simply improve and expand their well-established products. External pressures often stimulate a drive for renewal" [4]. Second, once a dominant design emerges it has the effect of enforcing or encouraging standardization so that production economies and/or other complementary economies can be sought. Competition then tends to focus on cost and scale as well as on product performance. The dominant design can have a profound impact on the direction and rate of further technical advance and on the structure of competition. Third, there are linkages between product technologies, manufacturing process, corporate organization and strategy, and the relationships between manufacturers, distributors and customers. Finally the period of technological turbulence caused by an invading (discontinuous, disruptive) innovation is a challenge that calls for a "bold response by established firms. More often than not the boldness comes from upstart firms with no standing in the industry in change. ... Established, dominant firms are more attracted to incremental (sustaining) innovations than to radical innovations" [5].

### Organizational Change as a Response to Innovation

Michael Porter in The Competitive Advantage of Nations, notes that innovators are frequently outsiders to the existing industry. Where innovators are large firms, they are often new entrants to the industry from an established position in another industry [6]. Failing firms are often remarkably creative in defending their entrenched technologies, which sometimes reach unimagined heights of elegance in design and technical performance only when their demise is clearly predictable. A poignant medical example of this may be the technical elegance of many of the off pump coronary artery bypass techniques as a response to angioplasty and stent procedures for coronary artery occlusions. A large and powerful firm can often respond with great creativity in defense of its own products, while rarely exhibiting the creativity required to embrace new discontinuous or disruptive technologies that would require it to abandon the old concepts. Utterback notes that this is "primarily the result of the habits of mind, commitments and strategy, or patterns of behavior of the organization's elite" [7].

An even clearer understanding of this process emerges from the work of Clayton Christensen. His is a landmark study entitled The Innovator's Dilemma in which he demonstrated why many well managed companies fail [8]. He has developed insights to help prevent future organizations from falling into the same trap. Clearly cardiothoracic surgery in its purest form is a profession and not a business enterprise, and many surgeons would be offended at the analogy; however, there are lessons discovered by Christensen that apply to cardiothoracic surgery as well.

### Sustaining and Disruptive Technologies

It is Christensen who has described two main types of technological innovation which confront established business enterprises: "**sustaining technology**" with which these enterprises deal reasonably successfully, and what he calls "**disruptive technology**", which established organizations often fail to incorporate and manage. Sustaining technologies are those that foster improved product performance. In contrast, disruptive technologies are often very different from the mainstream technology rather than being an incremental improvement. Disruptive technologies usually will under-perform established products in mainstream markets at least when they are first introduced, but they have other features that a few new customers value. Cardiothoracic surgery as a discipline, like many businesses, has been quite successful over the past four decades in developing, harnessing, and incorporating sustaining technologies which include cardiopulmonary bypass, as well as new techniques, treatments, and methodologies which enhance the ability to do coronary artery bypass surgery. It is having difficulty, however, adapting to the newer more radical technologies in the field of cardiac care such as angioplasty, stents, and catheter based arrhythmia treatments, etc. Some of Christensen's business analogies clarify the reasons for these difficulties and make the responses to these disruptive technologies more predictable.

Christensen studied a list of leading companies in a variety of fields. Each of the examples he cited went on to fail when confronted with disruptive changes in technology and market structure. He uses examples from retail marketing, heavy equipment manufacturing, computers, and the steel industry to show that the principles he espouses are broadly applicable. One theme common to the business failures he studied was that "the decisions that led to failure were made when the leaders in question were widely regarded as among the best companies in the world. ... There is something about the way decisions get made in successful organizations that sows the seeds of eventual failure"[9].

Christensen finds that there is a strategically important distinction between sustaining technologies and those that are disruptive. "What all sustaining technologies have in common is that they improve the performance of established products along the dimensions of performance that mainstream customers in major markets have historically valued. ... Rarely have even the most radically difficult sustaining technologies precipitated the failure of leading firms" [10]. Cardiac surgery has a long successful track record for developing and successfully incorporating sustaining technologies into the practice, whether it is new techniques of bypass or cardioplegia, new operative techniques or new prosthetic devices. The complexity of cardiac surgery is astounding. Cardiothoracic surgeons have been able to progressively improve cardiac operations while decreasing the time to perform them and decreasing patient mortality and morbidity.

Christensen observes that disruptive technologies occasionally emerge: "innovations that result in worse product performance, at least in the near term." Ironically in the cases he studied it was disruptive technology that precipitated leading firms' failures. "Disruptive technologies bring to a market a very different value proposition than had been available previously. Generally, disruptive technologies underperform established products in mainstream markets, but they have other features that a few fringe (and generally new) customers value. Products based on disruptive technologies are typically cheaper, simpler, smaller, and frequently, more convenient to use" [11]. The most obvious disruptive technology, which cardiac surgeons face, is balloon angioplasty of coronary arteries. In its initial incarnation, angioplasty was fairly limited to proximal single vessel disease and resulted in a high rate of restenosis. Sometimes angioplasty failed dramatically, resulting in the need for emergency coronary artery bypass surgery. The procedure was developed by outsiders, in this case cardiologists and radiologists, who traditionally were not involved in therapeutic surgical type interventions, but whose practices had been previously confined to diagnosis and medical management. Angioplasty found a niche market because it avoided unpleasant surgery. Initially it was not viewed as much of a threat by the cardiac surgery establishment. Some even thought it might increase the volume of traditional cardiac surgery performed, which early-on proved to be the case.

Christensen's second observation may seem a bit more abstract to the non-business reader. It is that technologies sometimes can progress faster than market demand. In the effort to provide better products than their competitors and earn higher prices and margins, suppliers often "overshoot" their market [12]. Examples are the watch, or the camera or indeed the pacemaker, which has so many "bells and whistles" that consumers never use, never learn about, and never care about, so they use the product only in its simplest most rudimentary mode. A simpler, cheaper, less elegant alternative may be able to supply some of the market need. In a similar fashion, the disruptive technology improves over time, even if it never becomes as sophisticated as the mainstream technology. This allows disruptive technologies, which underperform today relative to what users in the market require, to become fully performance competitive in that same market tomorrow. To continue with a cardiac example, angioplasty even with the newest stent technology will probably never be as elegant, as long lasting or as safe as coronary artery bypass grafting with a mammary artery. Angioplasty, however, has become successful enough and safe enough to satisfy a large portion of the "market".

Christensen's final set of observations is that established companies using good management practices make their decisions using financial decision making tools. They almost always conclude that investing aggressively in disruptive technologies is not a rational financial decision for them to make. There are three reasons for this. "First, [because] disruptive products are simpler and cheaper, they generally promise lower margins, not greater profits. Second, disruptive technologies typically are first commercialized in emerging or insignificant markets. And third, leading firms' most profitable customers generally don't want, and indeed initially can't use, products based on disruptive technologies. By and large, the least profitable customers in a market initially embrace a disruptive technology. Hence, most companies with a practiced discipline of listening to their best customers and identifying new products that promise greater profitability and growth are rarely able to build a case for investing in disruptive technologies until it is too late"[13]. It is not immediately clear how this last set of observations directly relates to a medical field. It seems too closely aligned to business, profit margins, and effective management. What Christensen is saying which does apply, is that using good, sound management and decision making tools as a screen has frequently not allowed managers to recognize the importance of new radical technologies. These tools effectively screen disruptive technologies from consideration. Since there is something called a "first mover" advantage, the young aggressive upstart company (or medical specialty), which early on embraces the new technology, frequently ends up being the leader in the new field and displaces the older, more established firm. This is a scenario in pharmaceutical and medical technology firms that is fairly common.

### Organizational Forces

Christensen feels that every company in every industry works under "certain forces – laws of organizational nature – that act powerfully to define what that company can and cannot do. Managers faced with disruptive technologies fail their companies when these forces overpower them" [14]. Similarly, the professional forces faced by cardiothoracic surgeons impact on their ability to deal with disruptive technology and to come up with alternative organizational structures in order to deal with them.

Companies depend on customers and investors for resources. Established firms "stayed atop wave after wave of sustaining technologies (technologies which their customers needed), while consistently stumbling over simpler disruptive ones." The theory of resource dependence states that "managers may think they control the flow of resources in their firms, but in the end it is really the customers and investors who dictate how money will be spent because companies with investment patterns that don't satisfy their customers and investors don't survive." Well-managed companies have developed systems for killing ideas that their customers don't want. They find it difficult to invest in disruptive technologies – lower margin opportunities that their mainstream customers don't want – until these customers recognize a use for them, and by then it is too late [15]. Cardiothoracic surgeons are appropriately conservative and have been content with their effective procedures. It has been difficult for them to devote time, training and research resources into such non-traditional and disruptive technologies as angioplasty, percutaneous vascular grafts, radio-frequency arrhythmia ablation and probably many other technologies until outsiders have established them and cardiothoracic surgeons have lost the "first mover advantage".

Small markets don't solve the growth needs of large companies. "Disruptive technologies typically enable new small markets to emerge. ... Companies entering these emerging markets early have significant first-mover advantages over later entrants. And yet as these companies succeed and grow larger it becomes progressively more difficult for them to enter the even newer small markets destined to become the large ones of the future" [16]. There is a mathematical reason for this. Investors expect a certain percentage return on their investment. A small company can invest in a small market and a relatively small profit will satisfy this percentage. The small returns from a new market would not satisfy the growth requirements for a large company. Stated simply, "while a $40 million company needs to find just $8 million in revenues to grow at 20 percent in the subsequent year, a $4 billion company needs to find $800 million in new sales. No new markets are that large. ... The larger and more successful an organization becomes, the weaker the argument that emerging markets can remain useful engines for growth"[17].

In an analogous way, cardiothoracic surgery was founded as a broad, anatomically based surgical specialty. Cardiothoracic surgeons, however, increasingly concentrated on a few high volume, high intensity, high cost procedures with excellent financial return. Even when they were innovative leaders in new disruptive technologies such as fiberoptic bronchoscopy, cardiac pacemakers, and insertion of intra-aortic balloon pumps, they were content to relinquish leadership roles in these areas to others. It made good business sense to concentrate on those procedures that yielded a higher marginal return on their investment of time and energy. They were too large an enterprise to fool with these "small markets" and so they yielded the "first mover advantage" to other newer players such as pulmonologists, intensivists, anesthesiologists and cardiologists. This is the same type of rationale employed by the managers of large currently successful companies, even when their own research and development personnel may be well aware of and even have helped to develop some of these new technologies. The evidence is strong that formal and informal resource allocation processes make it very difficult for large organizations to focus adequate energy and talent on small markets, even when logic says they may be big markets someday.

Christensen's next principle is that markets that don't exist can't be analyzed [18]. Sound market research and good planning followed by execution according to that scheme are the hallmarks of good business management. Disruptive technologies, by definition are new and immature. It is impossible to analyze where they will lead or to know their ultimate impact. Yet it is in disruptive technologies where there is such an important first mover advantage. Companies whose investment processes demand quantification of market sizes and financial returns before they can enter a market get paralyzed or make serious mistakes when faced with disruptive technologies. Here is where medicine has a great advantage over business. Its traditions emphasize the value of basic research, the kind that has no goal but scientific knowledge, which may or may not lead to a technological or medical breakthrough. Physicians, through their foundations and institutions, support this type of research, which has resulted in tremendous societal benefit. Even here there is a danger, as research resources tighten. Some grant processes award money to researchers for work already done, and support "established labs" at the expense of researchers with new "thinking out of the box" concepts. It is extremely important to avoid the pitfall of having research and technological efforts over-managed.

### Organizational Paradigms and Performance

This leads into the next principle, which is that an organization's capabilities also define its disabilities. Organizations have capabilities that exist independent of the people who work within them. These include their processes and their values. In business it is these processes and values that cause employees to prioritize projects to develop "high-margin products" and to reject projects that develop "low-margin products" [19]. In cardiac surgery, processes and values may lead to rejection of technologies viewed as "too medical" in favor of more traditional surgical approaches involving cutting and sewing". An example might be the general surgeons nearly missing the boat on laparoscopic surgery. They were very slow in embracing it, possibly because it was viewed as primarily in the realm of the gynecologist or because it was viewed as too diagnostic a procedure for their tastes. The transcripts of many conferences and journals record pronouncements by distinguished academic surgeons stating that laparoscopic surgery was a fad, would never catch on, and had no place in modern surgical practice. Fortunately, this technological advance was championed by general surgeons in private practice and entered the mainstream in essence through the back door.

The final principle, which has already been alluded to, is that technology supply may not equal market demand. Although disruptive technologies initially can only be used in small markets remote from the mainstream, as they improve and catch on, they may become fully performance-competitive within the mainstream market against established products. This is because the pace of technological progress in products frequently exceeds the rate of performance improvement demanded by mainstream customers. The disruptive technology overtakes the performance demands of the mainstream market [20].

Christensen's thesis in a nutshell is that well-managed companies sometimes fail because "the very management practices that have allowed them to become industry leaders also make it extremely difficult for them to develop the disruptive technologies that ultimately steal away their markets." It is clear that failure to deal with disruptive technology stems from organizational failures and not from the lack of access or understanding of the technology itself. The organization's structure and practices prevent it from recognizing and investing in the disruptive technology until it is too late to compete successfully. Likewise, the profession of cardiothoracic surgery with its training programs, boards, organizations, sub specialization, and recertification has developed institutional blocks to the incorporation of disruptive technologies such as angioplasty, much as the industries outlined by Utterback and Christensen mastered sustaining technology but have failed to incorporate disruptive technologies.

### Technological Innovation and Cardiothoracic Surgical Practice

Thoracic surgery historically was an outgrowth of surgery, now known as general surgery. It was anatomically and physiologically based on the organs of the thorax. It was a broad based discipline that identified diagnostic and therapeutic modalities, mastered new procedures and successfully incorporated many sustaining technologies. Treatment of cardiac disease, (and similar arguments can be made for pulmonary disease), evolved along two separate parental lines from internal medicine to cardiology and from general surgery to thoracic surgery to cardiac surgery. This made sense when there was a distinction between "cerebral" diagnostic efforts and procedure oriented therapy. But this distinction has long vanished, as diagnoses now require interventions, and surgery can be performed not only with a knife but also less traumatically with a catheter or laser. Simultaneously, cardiac surgeons have been abandoning their broad based discipline to concentrate on the "dominant design" of procedures based on cardiopulmonary bypass, and specifically on coronary artery bypass grafting. Cardiothoracic surgeons have mastered the dominant design and in fact create more and more elegant sustaining technological refinements even as the market for their "product" evaporates. In the final analysis, the specialty has matured to the point that all cardiac diseases can be corrected, even if it requires transplantation therapy.

To deal with current and future disruptive technologies and, more importantly, to ensure a robust future which is not dependent on specific procedures will require major organizational change. There are two possibilities. The first is for cardiothoracic surgeons to revisit their historical roots as thoracic surgeons who were first general surgeons. The profession of cardiothoracic surgery can re-emphasize broad based learning in general surgery, vascular surgery, pulmonary surgery and cardiac surgery. This has a certain appeal since it was the way that many cardiothoracic surgeons were trained. However, this goes against prevailing trends among thoracic surgery educators to de-emphasize general surgery training, shorten residencies, and even in some circles to separate cardiac and general thoracic surgical training. The advantage of this more general approach is that it allows cardiothoracic surgeons to move into other fields and to be flexible as specific procedures and technologies go through their life cycle of popularity and obsolescence. It fails to deal, however, with the organizational complications caused by other surgical specialists staking claim to various parts of the surgical turf and defending it vigorously.

A second approach, which may be better, is to completely reorganize the care of patients with cardiac disease and similarly patients with pulmonary disease. The distinction between cardiologist and cardiac surgeon based on the historical difference between internal medicine and surgery is now largely obsolete. Rather than start as a surgical or medical resident, it may be better that cardiac practitioners start as such and train in a broad based environment which incorporates all aspects of cardiac disease including diagnosis, interventional radiology, interventional cardiology, electrophysiology and cardiac surgery. In six or seven years, residents could be fully functional in all aspects of cardiac care, although admittedly they would probably want to subspecialize. These new practitioners would no longer fear obsolescence. They would control their own destiny. No matter what treatments and procedures come and go, if it involved the heart this specialty would be the operative force. A similar specialty could be set up for pulmonary medicine. This type of training would develop the consummate practitioner, involved in all aspects of the patient's care.

There is a precedent for such an approach. The most successful surgical specialties today are those that are based on organ systems, which have no medical counterpart, such as orthopedics, ophthalmology, otolaryngology, and to a lesser extent urology. They have continued to manage the diagnosis, medical treatment and surgical treatment in their respective areas. There are subspecialists within these areas but their broad based training makes it easier, for example, for a surgeon to move from the practice of one orthopedic subspecialty to another as technology changes than to move say from cardiac surgery to cardiology. A new specialty encompassing all aspects of both cardiac surgery and cardiology would control the flow of its patients and its patient's management regardless of whether the treatment is "medical", "surgical", chemotherapeutic, etc. Practitioners of this new specialty need not fear either evolutionary or revolutionary change in their field because they would be organized in a way that allows adaptation to any technological change. They would be free to do what is right for their patients without concern about how technology might modify their practice, their referrals or their livelihoods.

The difficulty with this type of a solution would be the transitional period. Current practitioners of both cardiac surgery and cardiology would have to be at least partially re-trained. Such a solution would represent a major organizational challenge requiring the remaking and/or consolidating of boards, training programs, professional organizations and, most difficult, the paradigms in which surgeons and cardiologists think of themselves. It would require changes in reimbursement rules and involve the role of government, particularly Medicare. It would be possible, though difficult.

Other specialties have remade themselves, usually through the determined efforts and clout of their specialty boards. One example is the post tonsillectomy era during which otolaryngology nearly disappeared but reemerged as a dynamic specialty incorporating head and neck cancer surgery, oncology, audiology, otology, some aspects of medicine, and even plastic surgery. Another example is anesthesiology, which under the assault of "nurse anesthesia" has reemerged as a more dynamic specialty incorporating anesthesia/critical care and pain management. A final example is radiology, which has evolved from a purely diagnostic technologically based field into almost a surgical subspecialty emphasizing interventional procedures.

Rosalind Williams concludes, in her book Retooling, a Historian Confronts Technological Change, that "technological change forces a series of decisions about money and control. People and institutions adapt, but adaptation is neither automatic nor straightforward. ... The ideology of technological change implies that market forces have replaced historical forces. This is not so and will never be so. Historical change is not planned, nor is it avoidable. What drives it is more primal than teams or technology. Historical change happens when people's deepest feelings get involved, when they are confronted by a crisis, when they have to question their habits and assumptions – even cherished ones, because sometimes habits and assumptions are no longer sustainable no matter how much they are cherished. This kind of change calls upon human beings to assume responsibility and to act courageously" [21].

## Conclusion

Our history, traditions, and organizations may prevent radical change in the field of cardiothoracic surgery. We need to stand cautioned by the many industrial examples cited in the papers by Utterback and Christenson that suggest that those who cannot adapt will disappear, but the changes will nonetheless happen. It is better for us to be open-minded and flexible in our organizational structures so that we will control our own professional destiny. Based on the business models outlined here, it is my premise that cardiothoracic surgery is at the crossroads between sustaining and disruptive technological innovations. As a discipline, cardiothoracic surgery must now choose between adaptation and controllable change as opposed to stagnation and obsolescence.

## Competing interests

The author(s) declare that they have no competing interests.
